# Double aortic arch in Honduras: an unusual cause of dysphagia in an adult patient. A case report

**DOI:** 10.47487/apcyccv.v6i2.482.

**Published:** 2025-06-27

**Authors:** Skarleth Paola Bock Alvarado, Andrea Nicole López García, Mónica Isabel Yanes Oviedo, Haroldo Arturo López García

**Affiliations:** 1 Facultad de Ciencias Médicas, Universidad Nacional Autónoma de Honduras. Tegucigalpa, Distrito Central, Francisco Morazán, Honduras. Facultad de Ciencias Médicas Universidad Nacional Autónoma de Honduras Tegucigalpa, Distrito Central, Francisco Morazán Honduras; 2 Departamento de Cardiología, Instituto Nacional Cardiopulmonar, Tegucigalpa, Distrito Central, Francisco Morazán, Honduras. Departamento de Cardiología Instituto Nacional Cardiopulmonar Tegucigalpa, Distrito Central, Francisco Morazán Honduras; 3 Servicio de Cardiología, Hospital Honduras Medical Center, Tegucigalpa, Francisco Morazán, Honduras. Servicio de Cardiología Hospital Honduras Medical Center Tegucigalpa, Francisco Morazán Honduras

**Keywords:** Adult, Aortic Arch Syndromes, Cardiovascular Abnormalities, Vascular Rings, Adulto, Anillo Vascular, Anomalías Cardiovasculares, Síndromes del Arco Aórtico

## Abstract

Vascular rings represent less than 1% of congenital cardiovascular anomalies, with double aortic arch being the most common variant. It is typically diagnosed in infants, with respiratory symptoms in over 90% of cases. We present the case of a 31-year-old male patient with recurrent childhood respiratory infections and asthma, who presented with progressive dysphagia. Contrast-enhanced computed tomography angiography revealed a double aortic arch with right-sided dominance, and the esophagram revealed compression of the proximal third of the esophagus. A left posterolateral thoracotomy was performed with division of the distal left arch, division of the ligamentum arteriosum, adhesions release, and thoracic aorta reconstruction. Postoperative recovery was favorable, with complete resolution of symptoms. Double aortic arch is rarely diagnosed in adulthood. This case highlights its atypical presentation, with predominant gastrointestinal symptoms, and the importance of considering it in the differential diagnosis of dysphagia.

## RESUMEN

Los anillos vasculares representan menos del 1% de las anomalías cardiovasculares congénitas, siendo el doble arco aórtico la variante más frecuente. Generalmente, se diagnostica en lactantes, con síntomas respiratorios en más del 90% de los casos. Presentamos el caso de paciente masculino de 31 años con antecedentes de infecciones respiratorias recurrentes y asma en la infancia, quien consultó por disfagia progresiva. La angiotomografía contrastada evidenció un doble arco aórtico con predominio derecho, y el esofagograma reveló compresión del tercio proximal esofágico. Se realizó una toracotomía posterolateral izquierda con sección del arco izquierdo distal, división del ligamento arterioso, liberación de adherencias y reconstrucción de la aorta torácica. La evolución posoperatoria fue favorable, con resolución completa de los síntomas. El doble arco aórtico, raramente se diagnostica en la adultez. Este caso resalta su presentación atípica con síntomas digestivos predominantes, y la importancia de considerarlo en el diagnóstico diferencial de disfagia.

## Introduction

Currently, the prevalence of aortic arch malformations is estimated at 1-2% in the general population ^1^. Vascular rings account for less than 1% of congenital cardiovascular anomalies, with double aortic arch (DAA) being the most common type, representing 46-76% of cases ^2^^,^^3^. This anomaly was first described by Wolman in 1939 ^3^^,^^4^. DAA is considered the vascular malformation most frequently associated with congenital external compression of the central airway ^5^. Approximately 55% of patients requiring surgical repair for a vascular ring have a DAA; to date, no sex or racial predilection has been identified ^6^. DAA is frequently associated with the 22q11 microdeletion syndrome, also known as DiGeorge syndrome ^7^.

DAA results from the persistence of both the right and left fourth embryonic aortic arches, forming a complete vascular ring that encircles the trachea and esophagus ^1^. Incomplete regression of the right arch with persistence of the left arch leads to the formation of a DAA ^6^. Based on anatomical characteristics and their relationship to the trachea and bronchi, DAA can be subdivided into three categories: right-dominant arch (80%), left-dominant arch (10%), and balanced aortic arches (10%) ^3^^,^^4^^,^^6^^,^^8^.

Symptoms depend primarily on the patient’s hemodynamic status and the degree of tracheal and esophageal compression. Clinical presentation may range from severe neonatal respiratory failure, apneic episodes, wheezing, stridor, cyanosis, persistent cough, recurrent respiratory infections, misdiagnosed asthma, to intermittent dysphagia, choking, regurgitation, and growth delay ^3^^,^^4^^,^^6^. Imaging studies are the diagnostic method of choice. Echocardiography may be useful as an initial tool to identify aortic arch anatomy and detect associated intracardiac anomalies. However, imaging modalities such as computed tomography and magnetic resonance imaging are preferred for their high sensitivity (100%) in detecting vascular rings ^4^^,^^6^^,^^8^^,^^9^. Management of DAA depends largely on symptom severity. Conservative treatment, including dietary and lifestyle modifications, may be considered for patients with mild to moderate symptoms ^10^. Surgical repair remains the only definitive treatment to relieve tracheobronchial compression and resolve severe symptoms ^4^^,^^6^^,^^10^.

We present the clinical case of a 31-year-old male adult with a long-standing history of dysphagia as the main symptom, along with a childhood history of recurrent respiratory infections and asthma. He was diagnosed in adulthood with a right-dominant DAA based on contrast-enhanced computed tomography angiography. The purpose of this report is to highlight the importance of considering DAA in the differential diagnosis of dysphagia in adults. It aims to contribute to the timely recognition of atypical presentations and to promote a multidisciplinary approach that ensures appropriate management and the prevention of complications.

## Case report

A 31-year-old mestizo male from Tegucigalpa, Francisco Morazán, Honduras, presented with progressively worsening long-standing dysphagia, recently accompanied by intermittent dyspnea and stridor, which had not been previously investigated. His medical history was notable for recurrent respiratory infections and childhood bronchial asthma. On initial physical examination, his blood pressure was 110/70 mmHg, heart rate 80 beats per minute, respiratory rate 18 breaths per minute, and temperature 37 °C. Cardiopulmonary auscultation revealed no pathological findings.

**Figure 1:**
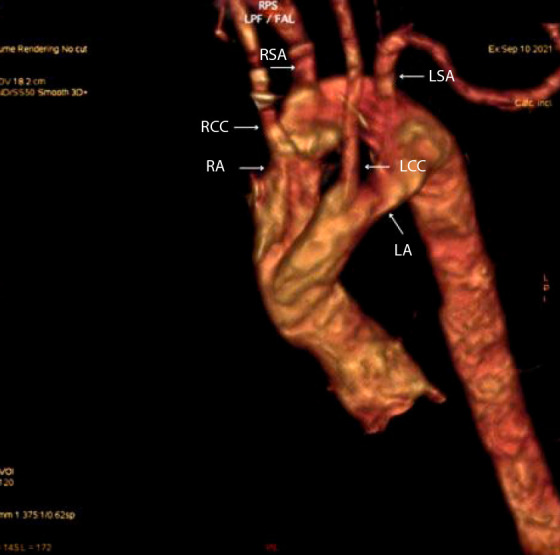
Contrast-enhanced chest computed tomography angiography with three-dimensional reconstruction, left anterolateral view. A complete double aortic arch forming a vascular ring is observed, with the right aortic arch (RA) being of larger caliber. The right (RCC) and left (LCC) common carotid arteries, as well as the right (RSA) and left (LSA) subclavian arteries, are identified. No brachiocephalic trunk is present. LA: left aortic arch

**Figure 2:**
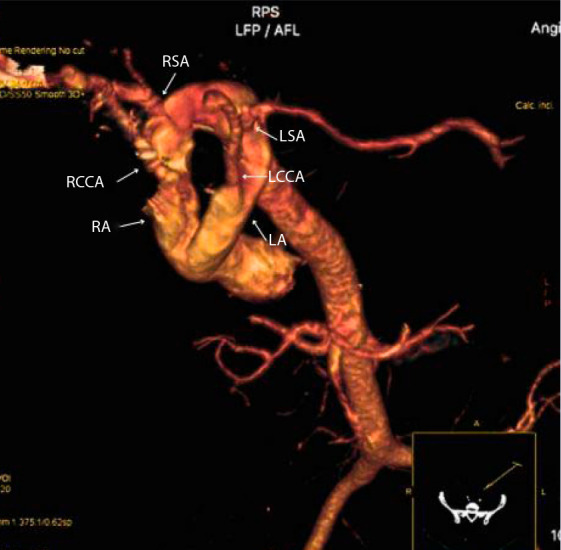
Contrast-enhanced chest computed tomography angiography with three-dimensional reconstruction, in a posterior inferior oblique view. The left aortic arch (LA) gives rise individually to the left common carotid artery (LCCA) and the left subclavian artery (LSA), while the right aortic arch (RA) gives rise to the right common carotid artery (RCCA) and the right subclavian artery (RSA).

As part of the initial diagnostic workup, a transthoracic echocardiogram was performed, which revealed a right aortic arch with no origin of the left subclavian artery, a left aortic arch with three supra-aortic branches, and suspicion of an aberrant subclavian artery. No septal defects or chamber dilatation were identified, and biventricular systolic function was preserved. Based on these findings, a contrast-enhanced computed tomography angiography was performed, revealing a DAA configuration: the right arch was dominant, measuring approximately 21 mm, and the left arch measured 14.5 mm. Both arches were patent and encircled the trachea and esophagus, forming a vascular ring with a junction of both components. No other cardiac or vascular anomalies were identified (Figures 1 and 2). Subsequently, an esophagram was performed, which showed a narrowing of the proximal third of the esophagus with an external impression suggestive of vascular ring compression.

Given the patient’s hemodynamic stability and the imaging confirmation of a DAA, an elective surgical procedure was scheduled for definitive correction. The operation was performed under general anesthesia, with right selective endobronchial intubation guided by bronchoscopy. Advanced hemodynamic monitoring was used, including electrocardiography, non-invasive and invasive blood pressure monitoring, capnography, measurement of the inspired oxygen fraction, and pulse oximetry. An arterial line was placed for continuous invasive pressure monitoring. The patient was positioned in the right lateral decubitus position, and a left posterolateral thoracotomy was performed with opening of the posterior mediastinum. A DAA was identified, with the right arch being dominant. The distal segments of both arches were carefully dissected, and the ligamentum arteriosum was divided and ligated. Vascular clamping of the thoracic aorta was then conducted at three sites: the right aortic arch, the left aortic arch, and the descending aorta. Total clamping time was 23 minutes. Cardiopulmonary bypass was not required, and therefore vascular cannulation was not performed. Resection of the distal segment of the left arch was carried out, followed by partial reconstruction of the thoracic aorta using an oval patch of reinforced expanded tissue measuring 1.8 x 1.8 cm, sutured with 4-0 Prolene. A two-layer suture technique was employed, reinforcing the stump of the left arch with the same prosthetic material for additional support. Hemostasis was meticulously confirmed.

Intraoperative blood loss was approximately 200 mL, and no blood products were required. Intercostal nerve block was performed using bupivacaine. A 28Fr Blake pleural drain was placed in the left pleural cavity, and surgical closure was completed in layers using standard technique. The patient was then transferred to the intensive care unit for appropriate monitoring and showed a favorable postoperative course without complications. He was discharged on postoperative day three with complete resolution of the initial symptoms.

## Discussion

Among vascular malformations classified as vascular rings, DAA is considered the most common type ^6^. It results from disruptions in the regression of the embryonic aortic arches, leading to the formation of a vascular ring that completely encircles mediastinal structures ^11^. The most frequent anatomical variant of DAA is the right-dominant arch, accounting for approximately 80% of cases ^3^^,^^6^^,^^8^^,^^12^. Our case corresponded to this most common presentation: a right-dominant aortic arch.

DAA presents with a broad spectrum of symptoms and, in some cases, remains asymptomatic. It is also diagnosed incidentally during evaluations performed for unrelated indications ^6^. Obstructive respiratory symptoms are reported in approximately 91% of cases, while digestive symptoms are present in about 40%. In most cases, symptoms manifest within the first six months of life ^3^^,^^11^^,^^12^. The present case is notable for the late onset of symptoms, which became evident in adulthood and were characterized by longstanding predominant dysphagia, recently accompanied by intermittent stridor and dyspnea. In Honduras, two previous cases of DAA have been reported, both diagnosed in infancy (at 7 and 24 months of age), with predominantly respiratory symptoms ^13^^,^^14^.

Although DAA tends to present earlier than other vascular rings, its diagnosis requires a high index of clinical suspicion due to its nonspecific symptoms, which necessitate ruling out other conditions. Computed tomography angiography is the diagnostic modality of choice, as it provides rapid, high-resolution imaging that enables precise anatomical evaluation ^3^^,^^11^. This condition is associated with other cardiovascular malformations in approximately 12% of cases ^9^. Ventricular septal defect is the most commonly identified intracardiac anomaly, followed by less frequent associations with atrial septal defect, patent ductus arteriosus, and tetralogy of Fallot ^11^^,^^15^. In the present case, no concomitant cardiovascular anomalies were found.

The cornerstone of treatment in symptomatic patients is surgical correction, with the primary aim of preventing potential airway development impairment and avoiding long-term complications. In asymptomatic patients, expectant management can be considered ^11^^,^^10^. Overall, the prognosis following surgical repair of DAA is excellent, with the most common adverse outcomes being persistent respiratory symptoms ^4^. Our patient had a favorable postoperative course, without complications, with appropriate follow-up, and currently shows complete resolution of symptoms.

In conclusion, DAA is a congenital vascular anomaly that, although typically diagnosed in infancy with respiratory symptoms, can present later in life with digestive symptoms such as dysphagia, posing a diagnostic challenge. Vascular rings should be included in the differential diagnosis of dysphagia in adults, particularly in patients with a history of childhood respiratory issues. In Honduras, the limited availability of neonatal cardiac screening and accessible imaging studies across most of the country contributes to delayed diagnoses, as illustrated by this case. This highlights the importance of expanding the quality and coverage of diagnostic resources in low- and middle-income countries. Early identification and appropriate surgical management are essential to prevent complications and improve the quality of life in patients with this condition.
